# Caregivers’ Profiles Based on the Canadian Occupational Performance Measure for the Adoption of Assistive Technologies

**DOI:** 10.3390/s22197500

**Published:** 2022-10-03

**Authors:** Francesco Della Gatta, Elisa Fabrizi, Franco Giubilei, María Dolores Grau, Carmen Moret-Tatay

**Affiliations:** 1 Department of Neuroscience, Mental Health and Sense Organs (NESMOS), Faculty of Medicine and Psychology, Sapienza University of Rome, 00185 Rome, Italy; 2 Escuela de Doctorado, Universidad Católica de Valencia San Vicente Mártir, 46001 Valencia, Spain; 3 Faculty of Psychology, Universidad Católica de Valencia San Vicente Mártir, 46100 Burjassot, Spain

**Keywords:** caregivers, COPM, assistive technology, satisfaction

## Abstract

The COPM (Canadian Occupational Performance Measure) is a tool that is based on the identification of self-perceived performance and satisfaction problems in the performance of occupations, allowing the creation of a hierarchy in the order of the interventions to be carried out, and speeding up the identification of the necessary AT (Assistive Technologies). Given the importance of the caregiver’s perception about their own performance in the design of AT, this research examines the caregiver’s profile through the COPM. A sample of 40 caregivers volunteered to participate in the study. A cluster analysis was carried out on the COPM scores. Two caregiver profiles were found in relation to the COPM measure, one with low scores on performance and satisfaction and another with high scores on both of these two variables. The main predictor was found to be the self-perception of performance. The structure was replicated through a hierarchical cluster analysis, where the role of caregivers was of interest. These results are relevant on both a theoretical and practical level.

## 1. Introduction

Caregivers vary from one to another in multiple ways. While the characteristics of care depend on the specific needs of the final client or relative in need, it is easy to underestimate the burden of care that the caregiver must cope with. This burden of care can be the cause of certain restrictions in the caregiver’s social and working life, particularly when informal caregivers are involved. Such restrictions might lead to changes in family relationships, exposing caregivers to high levels of stress [1]. Therefore, the onset of psychological disorders (usually anxiety and depression), as well as psychosomatic disorders, might occur [2,3]. The “burden of care” refers to the whole series of tasks that the caregiver must cover, such as the daily, physical, psychological, social, and economic needs of the client [4]. The burden varies according to the pathology and its severity, as well as the client’s age, gender, temperament, ethnicity, family history, and more. Additionally, there are other factors that may moderate the effects of caregiving, such as psychiatric history, personality, presence of chronic diseases, and socio-economic status [5,6]. As indicated by the current literature [7], understanding the close connection between client-related factors and the burden of caregivers appears to be a real challenge for several fields.

Emerging technologies are of interest in this regard. Several devices have been developed and are available to monitor medications or track clients to diminish the burden on caregivers. Moreover, these devices also provide real-time health information, which is of interest for health professionals. However, the literature has shown a need for further research and the evaluation of assistive technology (AT) in the caregiving field [8,9]. How people adopt technology is an issue of interest at all ages [10]. Further, specific ATs for caregivers and care recipients, especially those with dementia, should be examined for their potential to help the entire target population [11]. Collaborative design research that includes the input of caregivers’ profiles and their needs is of interest in order to mitigate the effects of the burden of care on their health.

Studies in the field have provided interesting examples of technology-based caregiving interventions [12]. However, to understand this adoption process, as well as to make caregivers’ mental health visible, it seems crucial to examine their needs. The concepts of sensors and smart homes are still evolving, and caregivers must be a prime target. In this work, the adoption of a theoretical framework based upon popular acceptance models in technology adoption is proposed. Likewise, the Technology Acceptance Model (TAM) is a theory that aims to model users’ acceptance of the use of a technological tool. In short, this classic model tries to examine how users accept and might use technology, paying special attention to the factors that influence their decision to adopt it. Authors such as Bagozzi [13] have pointed out that the TAM is a model that focuses on the individual user and essentially ignores developmental and social processes. Therefore, other implementations have been developed around the use of different external variables, offering further approaches. Among them, the Unified Theory of Acceptance and Use of Technology (UTAUT) proposes four constructs of interest in the field, namely: performance expectancy (PE), effort expectancy (EE), social influence (SI), and facilitating conditions (FCs) [14]. In this context, a study examined the factors that affect caregivers’ perceptions of the acceptance of wearable devices by patients with dementia, therefore extending the UTAUT model. The study concluded that SI, FCs, and EE have statistically significant effects on the behavior intention of a patient with dementia, in using healthcare wearable devices from their perspective. While the results look promising on client adoption, the direct literature on caregiver adoption is more limited.

It should be noted that caregivers must be supported and protected, which is not only in their own interest, but also in the interest of the client [9]; otherwise, they might become future patients themselves. Moreover, when caregivers adopt assistive technologies, they may also be an example to the client in enabling their adoption of the technology, as suggested by theoretical models (such as UTAUT) in relation to the effects of social influence. A systematic review on caregivers’ telehealth adoption identified a total of six categories for intervention, which are described as follows: education, consultation, psychosocial/cognitive behavioral therapy, data collection and monitoring, clinical care delivery, and social support [15]. While most of these categories are focused on caregiver activities, variables such as perceived effectiveness, usefulness, and acceptability perception were not addressed [16]. 

According to the literature, the assessment of cognitive status and functional autonomy could be considered discrete proxy indicators of distress [17]. Consequently, these parameters could be useful in identifying caregivers who are most in need of assessment and/or intervention. A piece of research highlighted the need for a holistic view, without losing sight of the experiences of those who are personally involved in the care [18]. This is also of interest for planning interventions, not only for treatment in the strict sense but also with the support of caregivers. Therefore, the therapeutic team should not forget to “support the supporters” during the long and eventful course of the disease.

Although there are different tools that have tried to measure aspects related to mental health, or the ability of caregivers to adopt technology, the caregiver’s self-perceptions in performance, to the best of our knowledge, are less explored under tools of reference such as the Canadian Occupational Performance Measure (COPM) [19]. In this way, the COPM is an evidence-based outcome measure designed to capture the self-perception of performance in everyday living over time. This Canadian approach for the practice of occupational therapy is named and framed as client-centered, and the COPM seeks to provide an answer as to how the occupational therapy intervention process should be in order to be consistent with person-centered practice. Although this measure has been mainly used with clients, its use with caregivers is less common, being, in most cases, related to clients who cannot provide this information due to an advanced state of cognitive impairment. Nevertheless, the COPM has demonstrated its flexibility and adaptability to different situations, clients, settings, and purposes [20]. The COPM is a tool that is based on the identification of self-perceived performance and satisfaction problems in the performance of occupations. Occupations are self-representative activities, important for self-realization and motivation. This latter aspect also makes occupations an indispensable means for an effective and efficient rehabilitation in economic terms. The COPM starts from a semi-structured interview that aims to identify all the performance problems in the occupation, in terms of taking care of the client. The use of the COPM for caregivers aims to help understand how to better facilitate the intervention of the only non-healthcare figure present, sometimes even 24 h a day, in the life of the patient. It allows us to create a hierarchy in the order of the interventions to be carried out, to speed up the identification of the necessary AT, e.g., devices for lifting people, tracking in case of wandering, safety in the kitchen and bathroom, and reducing the energetic impact of the activities to be carried out. Moreover, it can help us to better understand the process of AT adoption, thus highlighting new AT and better implementing the existing ones. Given the importance of the caregiver’s perception in the design of AT, this work examines the caregiver’s profile in terms of the COPM. Thus, according to the previous literature, it is hypothesized that different profiles emerge in terms of the COPM scores, and self-perception of performance is the main predictor for these profiles. If such profiles emerge, this information should be considered in the development of care devices.

## 2. Materials and Methods

### 2.1. Participants

A sample of 40 caregivers participated in the study, with a mean age of 55.95 (SD = 15.49) and an age range from 27 to 83 years old. A total of 75% were women. In terms of the time of caregiving performed, the mean amount of time was 4.95 (SD = 4.04). With regard to education, a total of 11.8% had studied for at least 5 years, 22.10% had studied for between 5 and 8 years, 35.3% had studied for between8 and 13 years and 30.9% had studied for more than 13 years. The client suffered from Alzheimer’s disease in 60% of the cases, 25% from Huntington’s Chorea and 15% from Multiple Sclerosis. Lastly, the caregiver’s role was defined as follows: 7.5% for Children, 37.5% for partners, 17.5% for relatives, and 37.5% for others (i.e., formal caregivers).

All Alzheimer’s and Huntington’s Chorea participants had moderate impairment according to the Mini–Mental State Examination.

### 2.2. Procedure and Ethics

This research took place at Sant’Andrea Hospital Rome (Italy). Those who wished to participate in the study received the information sheet and the informed consent form stating that participation was voluntary, and that the participant had the right to withdraw from the study at any time. Through an individual interview, variables such as age, level of education, the COPM, and medical history referred by the participant (to control for possible confounding variables) were collected. The inclusion criterion was being a caregiver. Exclusion criteria were as follows: not being fluent in the language of the research, suspected or reported cognitive impairment, and any other pathology that could influence the responses or the capacity to consent to the study. The session lasted around 35 min for each participant.

The study was carried out in accordance with the Declaration of Helsinki (ethical code of registration 6853). The participants received an informative form on the goals of the current research. Written, informed consent was compulsory. The participants were volunteers and were not coerced; they were free to leave at any time.

### 2.3. Materials

After collecting the personal data of interest, and maintaining anonymity, the COPM was employed [19]. During the COPM interview the areas that presented more occupational performance problems emerged. This information is crucial in order to select an adequate and personalized AT, depending on the area and/or the user. The COPM was developed by Law, Baptiste, Carswell, McColl, Polatajko and Pollock in 1990, and has been shown to be a validated and standardized tool. This measure can be divided into three subfactors that depict optimal psychometric values: importance (α = 0.66), performance (α = 0.84), and satisfaction (α = 0.80).

### 2.4. Design

This is an exploratory study under a cluster analysis. After descriptive analyses, a two-step cluster analysis was carried out on the COPM variables that were reported by caregivers. The probability test based on the Schwarz Bayesian Inference Criterion (BIC) was used to select the number of clusters. 

## 3. Results

**Figure 1:**
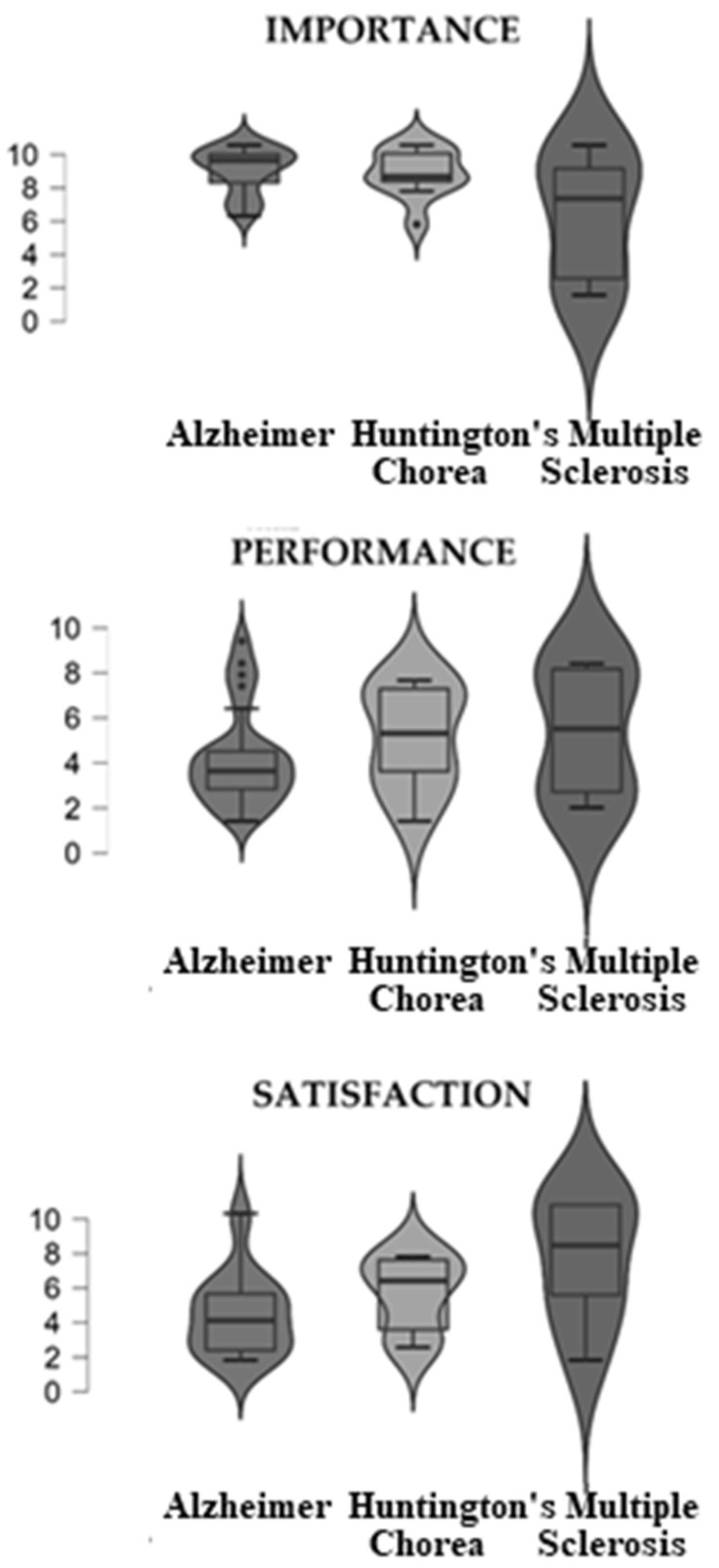
Box plots on COPM variables across pathologies.

Descriptive analyses were carried out on the variables of interest, as depicted in Figure 1 and Table 1. After a Kruskal–Wallis test, for a non-parametric approach, statistically significant differences were found on satisfaction scores in the COPM. Moreover, post hoc analyses were carried out as described in Table 2. In this case, this result suggested that caregivers of people affected by Alzheimer’s disease referred to lower levels of satisfaction than other groups.

An exploratory two-stage cluster analysis was performed to identify the number of clusters in the caregivers’ COPM scores. Likewise, the Schwarz Bayesian Inference Criterion (BIC) was also employed. We used the COPM scores to select the lowest BIC value in the different estimated models, in this case for two clusters. After the analysis, 100% of the cases were included, and the size ratio was optimal, with a value of 2.08. Two groups were formed with 32.5% of the cases as Group 1 and 67.5% for Group 2. 

**Figure 2:**
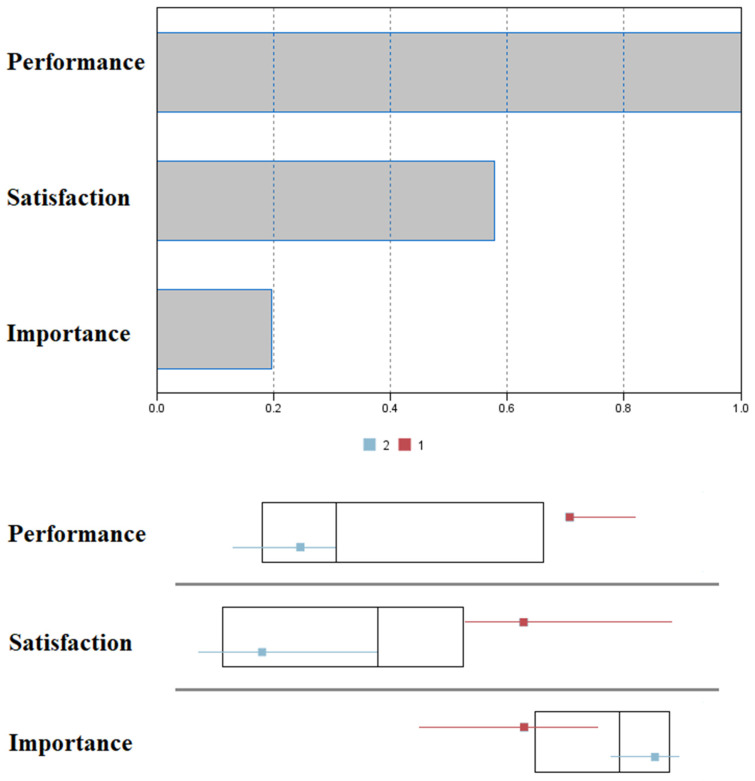
Box plots on cluster groups (**bottom**) and the importance of the predictors (**top**).

Figure 2 depicts a comparison between cluster groups, where performance and satisfaction were the most important variables. Comparisons were made across these new profiles. No statistically significant differences were found in terms of the time of caregiving across cluster groups (all *p* > 0.05 under *t*-test), nor across gender (under χ^2^ test). Differences across roles and clusters, however, reached the statistical significance level: χ^2^_(3)_ = 40; *p* < 0.01. 

**Figure 3:**
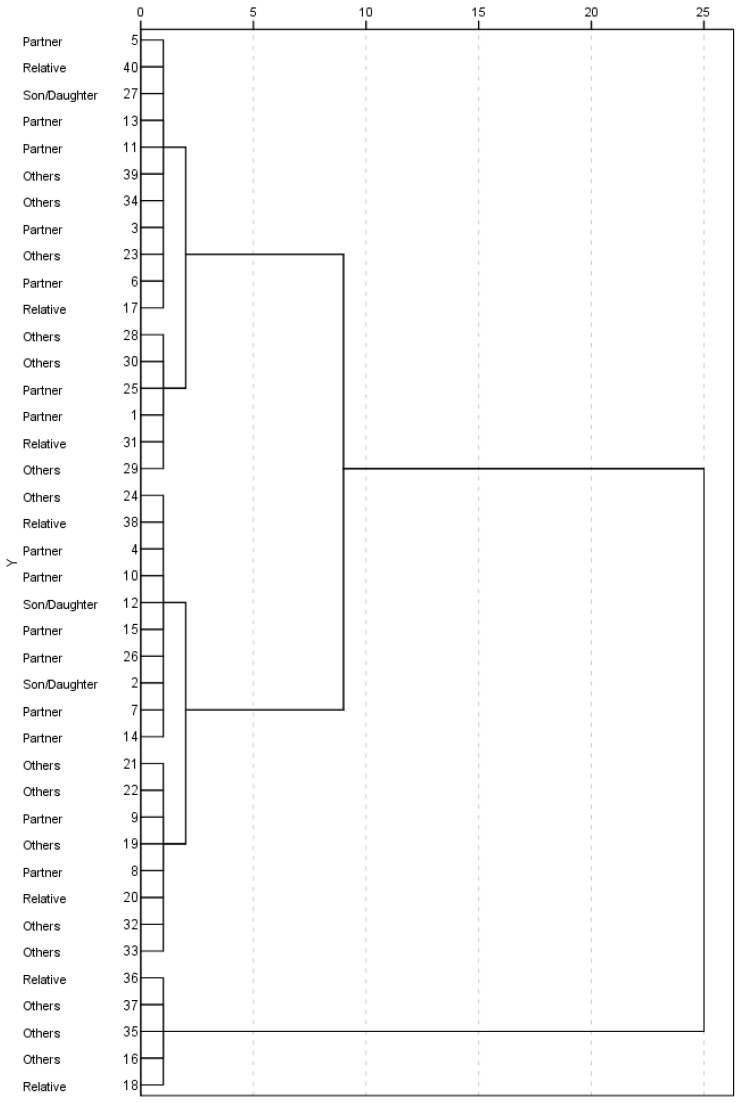
Tree diagram regarding satisfaction COPM and role scores in hierarchical cluster analysis.

Lastly, the proposed two-stage cluster analysis was replicated with a hierarchical cluster, as depicted in Figure 3. The structure is presented in a tree diagram format, which is chosen to help illustrate the groupings of the participants. Two large groups are presented, consistent with the previous analysis, except for two subjects, where differences in roles can be distinguished. 

## 4. Discussion

According to the literature, there are relatively few studies examining how caregivers use technology, e.g., fall alert technology, in their daily lives, or how such experiences may affect their own safety and wellbeing [20]. The purpose of this work was to study the different caregiver profiles through the COPM, and its application for AT. First, it should be noted that the COPM showed optimal psychometric properties in caregivers, supporting previous theories about its flexibility in use [21]. On the other hand, among its subfactors, importance was the least relevant and performance was the most important when describing the profiles. This would support the importance of assessing self-perceptions in caregiving performance, where occupational therapists and support devices play a relevant role for this process to occur correctly [22,23].

Two clusters were found after analysis, one with high satisfaction and performance perception scores, and another with low scores. These clusters were independent of the time of caregiving and the caregiver’s own age. However, the role was different, with more couples in cluster two having worse scores. This structure was replicated under a hierarchical cluster in terms of satisfaction across roles, as depicted in the tree diagram. Moreover, these clusters appear to be independent of the gender of the caregivers, which is a very controversial aspect in the literature [8,24]. However, we do not consider this result conclusive, and caution is advised here, given the fact that more women participated in the study than men. Thus, there is a potential bias toward conclusions in this direction. One should bear in mind that women tend to be overrepresented in this type of study, given their higher percentage of participation as caregivers in this field.

The cluster analysis showed that self-perception and satisfaction were the variables of main interest. In relation to the models on technology adoption, and more specifically the UTAUT model, it can be observed that the variables on caregivers differ from those on clients when making comparisons between current results and the previous literature. In other words, the perception of performance seems to be clearly related to caregiver satisfaction in current results, but one should bear in mind that this variable was not as relevant in previous studies for clients [16]. On the other hand, it should be noted that previous studies claimed that dysfunctional thoughts related to caregivers’ performance may affect their satisfaction with care as well as their burden perception [25]. This result is supported by the previous literature, which highlights the role of internal variables for health promotion in the caregiver context [26,27]. Moreover, one should bear in mind that, according to the literature, satisfaction is related to underlying aspects of mental health [28]. The mental health problems experienced by caregivers, due to the burden of care, are well known. Therefore, a low self-perception of performance could result in low satisfaction scores. At a theoretical level, research in this area may help to implement theoretical models of caregiving (e.g., stress models in the field) [29,30]. On a more applied level, these profiles are of interest for nurses and occupational therapists, among other healthcare workers, and particularly for AT design [6,7,31]. 

On the other hand, some specific constraints on the current study should be noted. First, the type of sampling was not selected using a random procedure, and sampling was incidental. This could lead to small biases in the generalization of the results. Secondly, the use of the COPM is less common in caregivers versus clients; nevertheless, the results seem promising in this field, as also supported by the previous literature [32]. Likewise, future lines of research should conduct both direct and systematic replications to unearth new evidence regarding the nature of these two profiles. Profiling caregivers through the COPM could direct research toward the development of specific AT techniques aimed at achieving certain objectives, such as more user-friendly interfaces or materials with particular characteristics (e.g., waterproof, light, or impact resistant). An interesting follow up of this study could be profiling through the activities with specific occupational performance problems, as indicated by caregivers. In summary, it is considered that the designers of AT to reduce the burden on caregivers should not forget the effects of self-perceptions, where the COPM measure could be of interest. It is alarming that if caregivers perceive their performance as poor then their satisfaction will drop, as well as the adoption of other devices. To the best of our knowledge, this is the first paper to employ this measure in caregivers to show evidence in the design of AT and we expect research to continue in this direction.

## 5. Conclusions

The main conclusions can be defined as follows: Two caregiver profiles were found in relation to the COPM, one with low scores on performance and satisfaction variables, and another with high scores on these two variables. The variable of importance was not relevant in the analysis. Further, the type of role varied from one cluster to another. These results are of interest, at both theoretical and applied levels, in the implementation of AT.

## Figures and Tables

**Table 1:** Descriptive analyses on the COPM variables across pathology and the Kruskal–Wallis test.

COPM	Pathology	Mean	SD	*p*
Importance	Alzheimer	8.52	1.37	0.29
	Huntington’s Chorea	8.34	1.46	
	Multiple Sclerosis	5.70	4.00	
	Total	7.70	2.12	
Performance	Alzheimer	3.82	2.18	0.55
	Huntington’s Chorea	4.72	2.27	
	Multiple Sclerosis	4.97	3.03	
	Total	4.74	2.36	
Satisfaction	Alzheimer	3.64	2.36	0.04
	Huntington’s Chorea	4.89	2.16	
	Multiple Sclerosis	6.82	3.78	
	Total	4.90	2.51	

**Table 2:** Dunn’s post hoc comparisons in satisfaction across pathologies *.

Comparison	z	W_i_	W_j_	p	p_bonf_	p_holm_
1–2	−1.686	16.938	24.350	0.046	0.138	0.092
1–3	−2.138	16.938	28.333	0.016	0.049	0.049
2–3	−0.661	24.350	28.333	0.254	0.763	0.254

* Note: 1 = Alzheimer; 2 = Huntington’s Chorea; 3 = Multiple Sclerosis.

## Data Availability

Data are available upon request.
